# Living donor liver transplantation using sensitized lymphocytotoxic crossmatch positive graft

**DOI:** 10.1007/s00535-012-0530-2

**Published:** 2012-02-11

**Authors:** Taku Aoki, Yasuhiko Sugawara, Michiro Takahashi, Yoshikuni Kawaguchi, Junichi Kaneko, Noriyo Yamashiki, Sumihito Tamura, Kiyoshi Hasegawa, Kouki Takahashi, Norihiro Kokudo

**Affiliations:** 1 Hepato-Biliary-Pancreatic Surgery Division, Department of Surgery, University of Tokyo, Tokyo, Japan; 2 Artificial Organ and Transplantation Division, Department of Surgery, University of Tokyo, 7-3-1 Hongo, Bunkyo-ku, Tokyo, Japan; 3 Organ Transplantation Service, University of Tokyo, Tokyo, Japan; 4 Department of Transfusion Medicine, Graduate School of Medicine, University of Tokyo, Tokyo, Japan

**Keywords:** Anti-CD25 antibody, Lymphocytotoxic crossmatch, Living donor

## Abstract

We describe a successful living donor liver transplantation (LDLT) using a lymphocytotoxic crossmatch highly positive graft. A 41-year-old woman with alcoholic liver cirrhosis was referred as a potential candidate for LDLT, and her husband was willing to donate his partial liver. As the T-lymphocytotoxic crossmatch titer was over 10,000×, the patient was first infused with rituximab for preoperative desensitization, and then five rounds of plasmapheresis were performed. After the third plasmapheresis, the lymphocytotoxic crossmatch test was negative. A left liver graft including the caudate lobe was implanted, and anti-CD25 antibody (basiliximab) was administered on postoperative days 1 and 4. The postoperative course was uneventful except for an episode of mild acute cellular rejection on postoperative day 27. Although the impact of a lymphocytotoxic crossmatch-positive liver graft on acute cellular rejection and graft survival in LDLT remains controversial, perioperative desensitization may provide benefits when using a highly sensitized liver graft.

## Introduction

Renal transplantation rates are low among patients highly sensitized to human leukocyte antigen (HLA) because of the high rate of antibody-mediated rejection and subsequent graft loss. It was recently reported, however, that preoperative desensitization using an anti-CD 20 antibody (rituximab) and intravenous immunoglobulin improved transplantation rates in patients highly sensitized to HLA [1]. In contrast, the significance of a positive lymphocytotoxic crossmatch in living donor liver transplantation (LDLT) is controversial. Successful LDLT using a liver graft in which the lymphocytotoxic crossmatch was highly positive is reported.

## Case report

The recipient was a 41-year-old woman with end-stage liver disease due to alcoholic liver cirrhosis (model for end-stage liver disease score 21). At the age of 20, she was gravida one, para one. She was considered a candidate for liver transplantation because of repeated episodes of encephalopathy. Because of the severe shortage of cadaveric donor grafts in Japan, we planned an LDLT, and her husband was willing to donate his partial liver. The ABO blood type was identical, but the T lymphocytotoxic crossmatch titer was over 10,000× and the B lymphocytotoxic crossmatch titer was 128× (complement method with the dilution technique according to the standard National Institutes of Health technique) [2]. In addition, an examination of anti-HLA antibodies using fluorescent microspheres revealed that the recipient had donor specific antibodies (B51 and B52). The number of HLA mismatches was three. After obtaining written informed consent from the patient and donor and the approval of the intra-institutional committee, we proceeded to the preoperative preparations.

For preoperative desensitization, the patient was first infused with rituximab 2 weeks before the scheduled surgery (due to a catheter-associated infection, however, the operation was postponed and LDLT was performed 21 days after initiation of the rituximab therapy). As the antibody to hepatitis B core antigen was positive, entecavir (0.5 mg/day) was administered for 3 weeks preoperatively to prevent a possible hepatitis B virus breakthrough. Five rounds of plasmapheresis were performed. After the third plasmapheresis, the lymphocytotoxic crossmatch test was negative, and was sustained as negative thereafter.

**Fig. 1:**
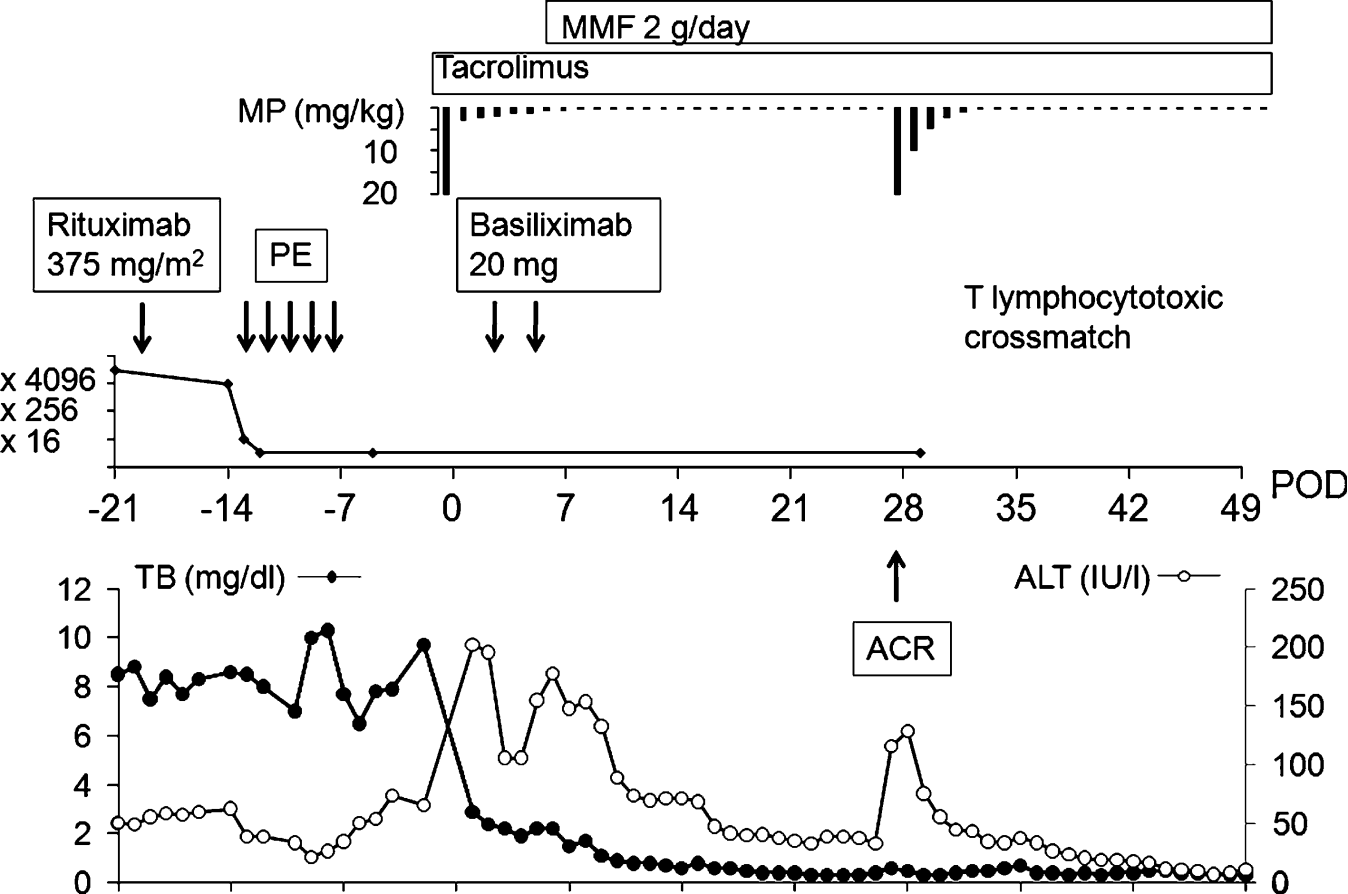
The clinical profile of the present patient. *ACR* acute cellular rejection, *ALT* alanine aminotransferase, *MMF* mycophenolate mofetil, *MP* methylprednisolone, *PE* plasma exchange, *TB* total bilirubin, *POD* postoperative day

A left liver graft including the caudate lobe was implanted. During the LDLT, splenectomy was performed. On postoperative days 1 and 4, 20 mg of anti-CD25 antibody (basiliximab) was administered in addition to the routine methylprednisolone and tacrolimus, as we were anxious about hyperacute rejection. Besides, mycophenolate mofetil (MMF; 2,000 mg/day) was started on postoperative day 7. The postoperative course was uneventful except for an episode of mild acute cellular rejection (Banff score 3) on postoperative day 27, which responded promptly to steroid recycle therapy. The liver biopsy specimen obtained at the time of the acute rejection showed mild infiltration of lymphocytes in the portal area and around the bile ducts. The clinical course of the recipient is summarized in Fig. 1. One year after the LDLT, the lymphocytotoxic crossmatch remained negative and the patient has been well with good graft function.

## Discussion

The impact of a lymphocytotoxic crossmatch-positive liver graft on acute cellular rejection and graft survival remains controversial, both in deceased donor liver transplantation [3, 4] and in LDLT [5–7]. Some institutions have reported significantly unfavorable outcomes in LDLT recipients with a positive lymphocytotoxic crossmatch [6, 7]. In contrast, our previous results [5] showed that if the titer is low (no more than 32×), a positive lymphocytotoxic crossmatch does not adversely affect the graft or survival in patients without desensitization. Although the significance of a quantitative assessment of the lymphocytotoxic crossmatch has not been reported, the high titer in our present patient led to the need for perioperative desensitization to prevent early graft loss due to antibody-mediated rejection. After considering the results in the present patient, we have settled the indication criteria for preoperative desensitization therapy at the titer of 1,000× (T lymphocyte crossmatch).

In this patient, the anti-donor antibodies were assumed to have arisen through pregnancy. Therefore, we applied preoperative desensitization using rituximab and plasmapheresis to reduce the high titer of preformed antibodies and B lymphocytes. As a result, the lymphocytotoxic crossmatch was negative after the 3rd plasmapheresis, and negativity was sustained thereafter. Preoperative desensitization using rituximab was introduced in ABO-incompatible LDLT in 2003 and has dramatically improved the outcomes of ABO-incompatible LDLT. The appropriate dosage of rituximab is still controversial, but many previous studies have reported the administration of 375 mg/m^2^ of rituximab 1–3 weeks before the transplant. Following these successful cases, we planned the administration of 375 mg/m^2^ (500 mg/body) of rituximab 2 weeks before the operation [8, 9]. In addition, we performed splenectomy during the LDLT. Splenectomy is also considered to be effective to reduce antibody production, as the spleen is the site of antibody production. After the operation, the suppression of T-cell function to prevent the initiation of T-cell-mediated antibody production was regarded as indispensable. We have routinely used tacrolimus and steroid as an immunosuppressive regimen, and in this particular patient, we added basiliximab (postoperative days [PODs] 1 and 4) and MMF. Mild acute cellular rejection occurred about 3 weeks after the LDLT, but response to the steroid recycle therapy was prompt, and the lymphocytotoxic crossmatch was negative during this episode.

In summary, we report a successful LDLT using a lymphocytotoxic crossmatch highly positive graft. Perioperative desensitization using plasmapheresis and rituximab may provide significant benefits for reducing anti-HLA antibodies.

## Abbreviations

HLA: Human leukocyte antigen
LDLT: Living donor liver transplantation
MMF: Mycophenolate mofetil
